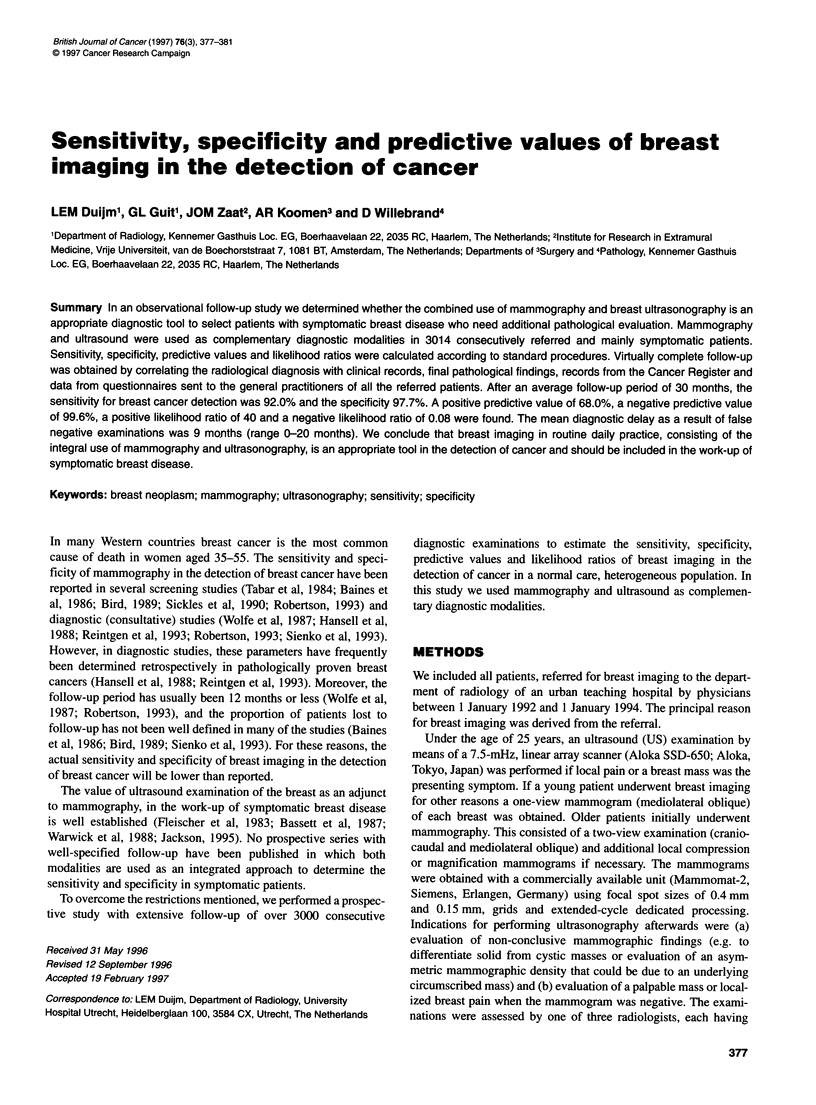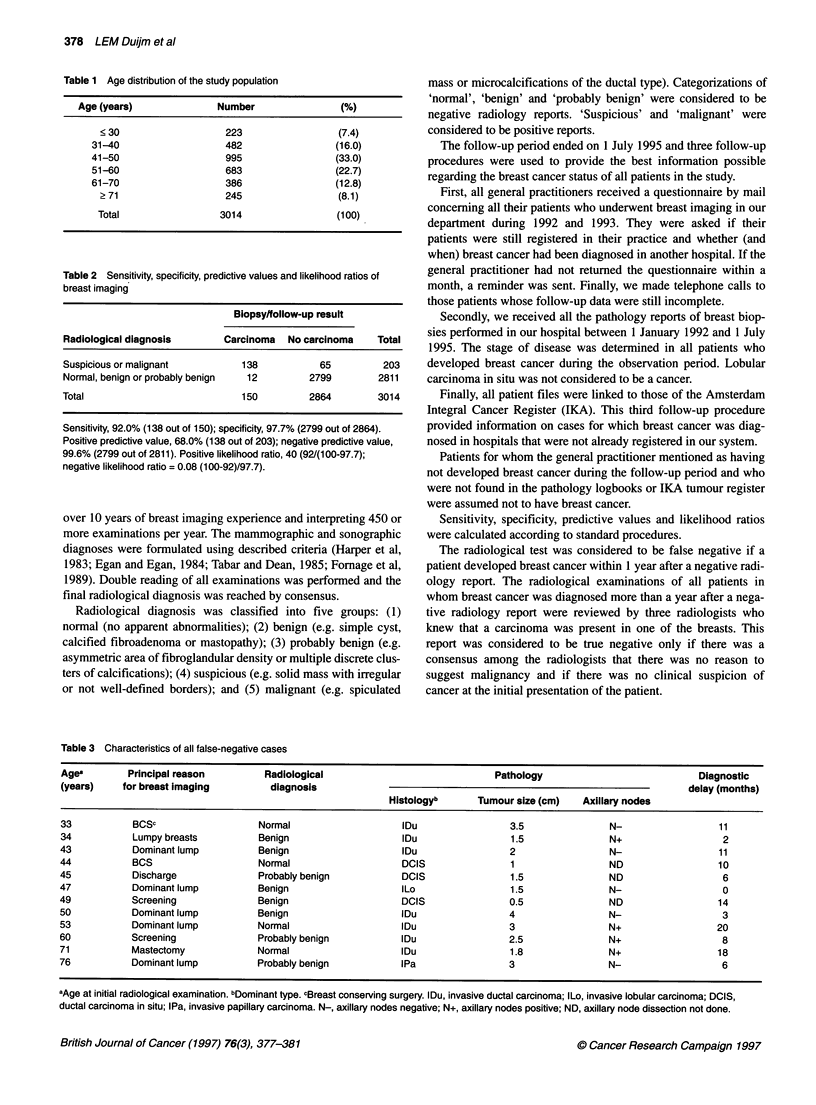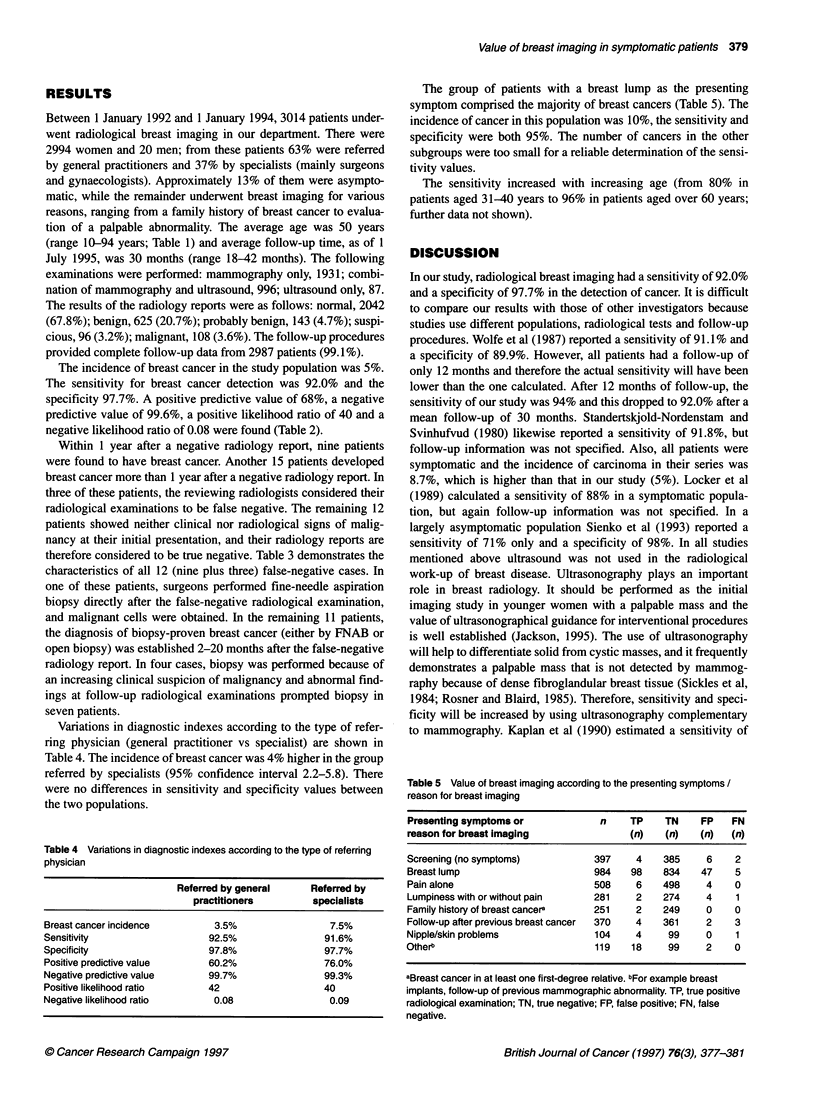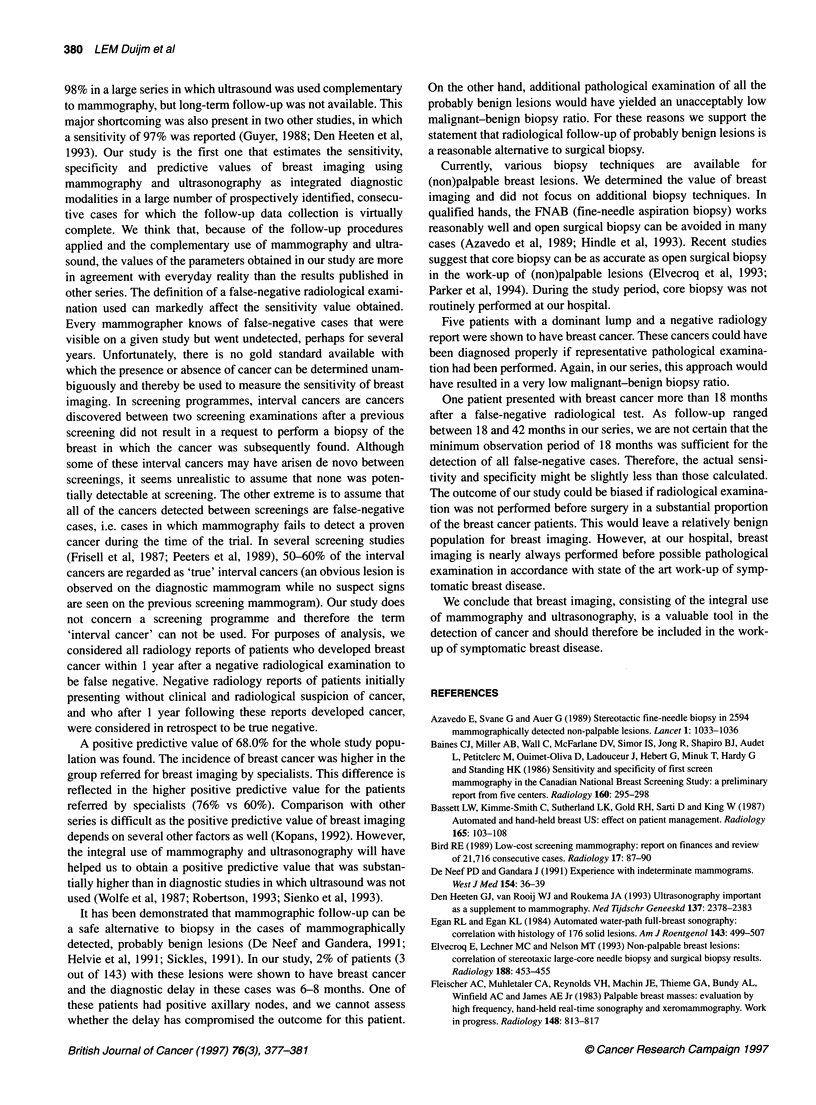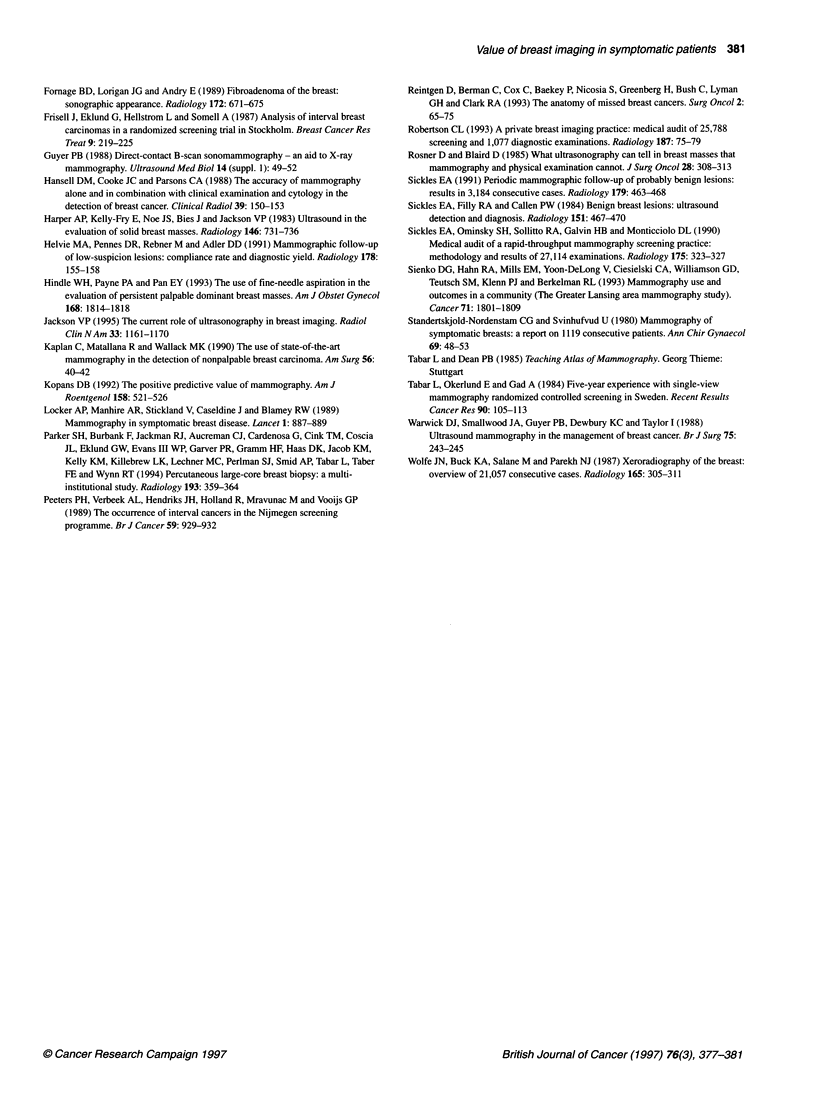# Sensitivity, specificity and predictive values of breast imaging in the detection of cancer.

**DOI:** 10.1038/bjc.1997.393

**Published:** 1997

**Authors:** L. E. Duijm, G. L. Guit, J. O. Zaat, A. R. Koomen, D. Willebrand

**Affiliations:** Department of Radiology, Kennemer Gasthuis Loc. EG, Haarlem, The Netherlands.

## Abstract

In an observational follow-up study we determined whether the combined use of mammography and breast ultrasonography is an appropriate diagnostic tool to select patients with symptomatic breast disease who need additional pathological evaluation. Mammography and ultrasound were used as complementary diagnostic modalities in 3014 consecutively referred and mainly symptomatic patients. Sensitivity, specificity, predictive values and likelihood ratios were calculated according to standard procedures. Virtually complete follow-up was obtained by correlating the radiological diagnosis with clinical records, final pathological findings, records from the Cancer Register and data from questionnaires sent to the general practitioners of all the referred patients. After an average follow-up period of 30 months, the sensitivity for breast cancer detection was 92.0% and the specificity 97.7%. A positive predictive value of 68.0%, a negative predictive value of 99.6%, a positive likelihood ratio of 40 and a negative likelihood ratio of 0.08 were found. The mean diagnostic delay as a result of false negative examinations was 9 months (range 0-20 months). We conclude that breast imaging in routine daily practice, consisting of the integral use of mammography and ultrasonography, is an appropriate tool in the detection of cancer and should be included in the work-up of symptomatic breast disease.


British Journal of Cancer (1997) 76(3), 377–381
© 1997 Cancer Research Campaign

### Sensitivity, specificity and predictive values of breast imaging in the detection of cancer

**LEM Duijm[1], GL Guit[1], JOM Zaat[2], AR Koomen[3] and D Willebrand[4]**

[1]Department of Radiology, Kennemer Gasthuis Loc. EG, Boerhaavelaan 22, 2035 RC, Haarlem, The Netherlands; [2]Institute for Research in Extramural Medicine, Vrije Universiteit, van de Boechorststraat 7, 1081 BT, Amsterdam, The Netherlands; Departments of [3]Surgery and [4]Pathology, Kennemer Gasthuis Loc. EG, Boerhaavelaan 22, 2035 RC, Haarlem, The Netherlands

**Summary** In an observational follow-up study we determined whether the combined use of mammography and breast ultrasonography is an appropriate diagnostic tool to select patients with symptomatic breast disease who need additional pathological evaluation. Mammography and ultrasound were used as complementary diagnostic modalities in 3014 consecutively referred and mainly symptomatic patients. Sensitivity, specificity, predictive values and likelihood ratios were calculated according to standard procedures. Virtually complete follow-up was obtained by correlating the radiological diagnosis with clinical records, final pathological findings, records from the Cancer Register and data from questionnaires sent to the general practitioners of all the referred patients. After an average follow-up period of 30 months, the sensitivity for breast cancer detection was 92.0% and the specificity 97.7%. A positive predictive value of 68.0%, a negative predictive value of 99.6%, a positive likelihood ratio of 40 and a negative likelihood ratio of 0.08 were found. The mean diagnostic delay as a result of false negative examinations was 9 months (range 0–20 months). We conclude that breast imaging in routine daily practice, consisting of the integral use of mammography and ultrasonography, is an appropriate tool in the detection of cancer and should be included in the work-up of symptomatic breast disease.

**Keywords:** breast neoplasm; mammography; ultrasonography; sensitivity; specificity

In many Western countries breast cancer is the most common cause of death in women aged 35–55. The sensitivity and specificity of mammography in the detection of breast cancer have been reported in several screening studies (Tabar et al, 1984; Baines et al, 1986; Bird, 1989; Sickles et al, 1990; Robertson, 1993) and diagnostic (consultative) studies (Wolfe et al, 1987; Hansell et al, 1988; Reintgen et al, 1993; Robertson, 1993; Sienko et al, 1993). However, in diagnostic studies, these parameters have frequently been determined retrospectively in pathologically proven breast cancers (Hansell et al, 1988; Reintgen et al, 1993). Moreover, the follow-up period has usually been 12 months or less (Wolfe et al, 1987; Robertson, 1993), and the proportion of patients lost to follow-up has not been well defined in many of the studies (Baines et al, 1986; Bird, 1989; Sienko et al, 1993). For these reasons, the actual sensitivity and specificity of breast imaging in the detection of breast cancer will be lower than reported.

The value of ultrasound examination of the breast as an adjunct to mammography, in the work-up of symptomatic breast disease is well established (Fleischer et al, 1983; Bassett et al, 1987; Warwick et al, 1988; Jackson, 1995). No prospective series with well-specified follow-up have been published in which both modalities are used as an integrated approach to determine the sensitivity and specificity in symptomatic patients.

To overcome the restrictions mentioned, we performed a prospective study with extensive follow-up of over 3000 consecutive diagnostic examinations to estimate the sensitivity, specificity, predictive values and likelihood ratios of breast imaging in the detection of cancer in a normal care, heterogeneous population. In this study we used mammography and ultrasound as complementary diagnostic modalities.

*Received 31 May 1996*
*Revised 12 September 1996*
*Accepted 19 February 1997*

*Correspondence to:* LEM Duijm, Department of Radiology, University Hospital Utrecht, Heidelberglaan 100, 3584 CX, Utrecht, The Netherlands

### METHODS

We included all patients, referred for breast imaging to the department of radiology of an urban teaching hospital by physicians between 1 January 1992 and 1 January 1994. The principal reason for breast imaging was derived from the referral.

Under the age of 25 years, an ultrasound (US) examination by means of a 7.5-mHz, linear array scanner (Aloka SSD-650; Aloka, Tokyo, Japan) was performed if local pain or a breast mass was the presenting symptom. If a young patient underwent breast imaging for other reasons a one-view mammogram (mediolateral oblique) of each breast was obtained. Older patients initially underwent mammography. This consisted of a two-view examination (craniocaudal and mediolateral oblique) and additional local compression or magnification mammograms if necessary. The mammograms were obtained with a commercially available unit (Mammomat-2, Siemens, Erlangen, Germany) using focal spot sizes of 0.4 mm and 0.15 mm, grids and extended-cycle dedicated processing. Indications for performing ultrasonography afterwards were (a) evaluation of non-conclusive mammographic findings (e.g. to differentiate solid from cystic masses or evaluation of an asymmetric mammographic density that could be due to an underlying circumscribed mass) and (b) evaluation of a palpable mass or localized breast pain when the mammogram was negative. The examinations were assessed by one of three radiologists, each having

**Table 1** Age distribution of the study population

| Age (years) | Number | (%) |
|---|---|---|
| ≤ 30 | 223 | (7.4) |
| 31–40 | 482 | (16.0) |
| 41–50 | 995 | (33.0) |
| 51–60 | 683 | (22.7) |
| 61–70 | 386 | (12.8) |
| ≥ 71 | 245 | (8.1) |
| Total | 3014 | (100) |

**Table 2** Sensitivity, specificity, predictive values and likelihood ratios of breast imaging

| | Biopsy/follow-up result | | |
|---|---|---|---|
| Radiological diagnosis | Carcinoma | No carcinoma | Total |
| Suspicious or malignant | 138 | 65 | 203 |
| Normal, benign or probably benign | 12 | 2799 | 2811 |
| Total | 150 | 2864 | 3014 |

Sensitivity, 92.0% (138 out of 150); specificity, 97.7% (2799 out of 2864). Positive predictive value, 68.0% (138 out of 203); negative predictive value, 99.6% (2799 out of 2811). Positive likelihood ratio, 40 (92/(100-97.7); negative likelihood ratio = 0.08 (100-92)/97.7).

over 10 years of breast imaging experience and interpreting 450 or more examinations per year. The mammographic and sonographic diagnoses were formulated using described criteria (Harper et al, 1983; Egan and Egan, 1984; Tabar and Dean, 1985; Fornage et al, 1989). Double reading of all examinations was performed and the final radiological diagnosis was reached by consensus.

Radiological diagnosis was classified into five groups: (1) normal (no apparent abnormalities); (2) benign (e.g. simple cyst, calcified fibroadenoma or mastopathy); (3) probably benign (e.g. asymmetric area of fibroglandular density or multiple discrete clusters of calcifications); (4) suspicious (e.g. solid mass with irregular or not well-defined borders); and (5) malignant (e.g. spiculated mass or microcalcifications of the ductal type). Categorizations of 'normal', 'benign' and 'probably benign' were considered to be negative radiology reports. 'Suspicious' and 'malignant' were considered to be positive reports.

The follow-up period ended on 1 July 1995 and three follow-up procedures were used to provide the best information possible regarding the breast cancer status of all patients in the study.

First, all general practitioners received a questionnaire by mail concerning all their patients who underwent breast imaging in our department during 1992 and 1993. They were asked if their patients were still registered in their practice and whether (and when) breast cancer had been diagnosed in another hospital. If the general practitioner had not returned the questionnaire within a month, a reminder was sent. Finally, we made telephone calls to those patients whose follow-up data were still incomplete.

Secondly, we received all the pathology reports of breast biopsies performed in our hospital between 1 January 1992 and 1 July 1995. The stage of disease was determined in all patients who developed breast cancer during the observation period. Lobular carcinoma in situ was not considered to be a cancer.

Finally, all patient files were linked to those of the Amsterdam Integral Cancer Register (IKA). This third follow-up procedure provided information on cases for which breast cancer was diagnosed in hospitals that were not already registered in our system.

Patients for whom the general practitioner mentioned as having not developed breast cancer during the follow-up period and who were not found in the pathology logbooks or IKA tumour register were assumed not to have breast cancer.

Sensitivity, specificity, predictive values and likelihood ratios were calculated according to standard procedures.

The radiological test was considered to be false negative if a patient developed breast cancer within 1 year after a negative radiology report. The radiological examinations of all patients in whom breast cancer was diagnosed more than a year after a negative radiology report were reviewed by three radiologists who knew that a carcinoma was present in one of the breasts. This report was considered to be true negative only if there was a consensus among the radiologists that there was no reason to suggest malignancy and if there was no clinical suspicion of cancer at the initial presentation of the patient.

**Table 3** Characteristics of all false-negative cases

| Age[a] (years) | Principal reason for breast imaging | Radiological diagnosis | Pathology | | | Diagnostic delay (months) |
|---|---|---|---|---|---|---|
| | | | Histology[b] | Tumour size (cm) | Axillary nodes | |
| 33 | BCS[c] | Normal | IDu | 3.5 | N– | 11 |
| 34 | Lumpy breasts | Benign | IDu | 1.5 | N+ | 2 |
| 43 | Dominant lump | Benign | IDu | 2 | N– | 11 |
| 44 | BCS | Normal | DCIS | 1 | ND | 10 |
| 45 | Discharge | Probably benign | DCIS | 1.5 | ND | 6 |
| 47 | Dominant lump | Benign | ILo | 1.5 | N– | 0 |
| 49 | Screening | Benign | DCIS | 0.5 | ND | 14 |
| 50 | Dominant lump | Benign | IDu | 4 | N– | 3 |
| 53 | Dominant lump | Normal | IDu | 3 | N+ | 20 |
| 60 | Screening | Probably benign | IDu | 2.5 | N+ | 8 |
| 71 | Mastectomy | Normal | IDu | 1.8 | N+ | 18 |
| 76 | Dominant lump | Probably benign | IPa | 3 | N– | 6 |

[a]Age at initial radiological examination. [b]Dominant type. [c]Breast conserving surgery. IDu, invasive ductal carcinoma; ILo, invasive lobular carcinoma; DCIS, ductal carcinoma in situ; IPa, invasive papillary carcinoma. N–, axillary nodes negative; N+, axillary nodes positive; ND, axillary node dissection not done.

© Cancer Research Campaign 1997

### RESULTS

Between 1 January 1992 and 1 January 1994, 3014 patients underwent radiological breast imaging in our department. There were 2994 women and 20 men; from these patients 63% were referred by general practitioners and 37% by specialists (mainly surgeons and gynaecologists). Approximately 13% of them were asymptomatic, while the remainder underwent breast imaging for various reasons, ranging from a family history of breast cancer to evaluation of a palpable abnormality. The average age was 50 years (range 10–94 years; Table 1) and average follow-up time, as of 1 July 1995, was 30 months (range 18–42 months). The following examinations were performed: mammography only, 1931; combination of mammography and ultrasound, 996; ultrasound only, 87. The results of the radiology reports were as follows: normal, 2042 (67.8%); benign, 625 (20.7%); probably benign, 143 (4.7%); suspicious, 96 (3.2%); malignant, 108 (3.6%). The follow-up procedures provided complete follow-up data from 2987 patients (99.1%).

The incidence of breast cancer in the study population was 5%. The sensitivity for breast cancer detection was 92.0% and the specificity 97.7%. A positive predictive value of 68%, a negative predictive value of 99.6%, a positive likelihood ratio of 40 and a negative likelihood ratio of 0.08 were found (Table 2).

Within 1 year after a negative radiology report, nine patients were found to have breast cancer. Another 15 patients developed breast cancer more than 1 year after a negative radiology report. In three of these patients, the reviewing radiologists considered their radiological examinations to be false negative. The remaining 12 patients showed neither clinical nor radiological signs of malignancy at their initial presentation, and their radiology reports are therefore considered to be true negative. Table 3 demonstrates the characteristics of all 12 (nine plus three) false-negative cases. In one of these patients, surgeons performed fine-needle aspiration biopsy directly after the false-negative radiological examination, and malignant cells were obtained. In the remaining 11 patients, the diagnosis of biopsy-proven breast cancer (either by FNAB or open biopsy) was established 2–20 months after the false-negative radiology report. In four cases, biopsy was performed because of an increasing clinical suspicion of malignancy and abnormal findings at follow-up radiological examinations prompted biopsy in seven patients.

Variations in diagnostic indexes according to the type of referring physician (general practitioner vs specialist) are shown in Table 4. The incidence of breast cancer was 4% higher in the group referred by specialists (95% confidence interval 2.2–5.8). There were no differences in sensitivity and specificity values between the two populations.

**Table 4** Variations in diagnostic indexes according to the type of referring physician

| | Referred by general practitioners | Referred by specialists |
|---|---|---|
| Breast cancer incidence | 3.5% | 7.5% |
| Sensitivity | 92.5% | 91.6% |
| Specificity | 97.8% | 97.7% |
| Positive predictive value | 60.2% | 76.0% |
| Negative predictive value | 99.7% | 99.3% |
| Positive likelihood ratio | 42 | 40 |
| Negative likelihood ratio | 0.08 | 0.09 |

The group of patients with a breast lump as the presenting symptom comprised the majority of breast cancers (Table 5). The incidence of cancer in this population was 10%, the sensitivity and specificity were both 95%. The number of cancers in the other subgroups were too small for a reliable determination of the sensitivity values.

The sensitivity increased with increasing age (from 80% in patients aged 31–40 years to 96% in patients aged over 60 years; further data not shown).

### DISCUSSION

In our study, radiological breast imaging had a sensitivity of 92.0% and a specificity of 97.7% in the detection of cancer. It is difficult to compare our results with those of other investigators because studies use different populations, radiological tests and follow-up procedures. Wolfe et al (1987) reported a sensitivity of 91.1% and a specificity of 89.9%. However, all patients had a follow-up of only 12 months and therefore the actual sensitivity will have been lower than the one calculated. After 12 months of follow-up, the sensitivity of our study was 94% and this dropped to 92.0% after a mean follow-up of 30 months. Standertskjold-Nordenstam and Svinhufvud (1980) likewise reported a sensitivity of 91.8%, but follow-up information was not specified. Also, all patients were symptomatic and the incidence of carcinoma in their series was 8.7%, which is higher than that in our study (5%). Locker et al (1989) calculated a sensitivity of 88% in a symptomatic population, but again follow-up information was not specified. In a largely asymptomatic population Sienko et al (1993) reported a sensitivity of 71% only and a specificity of 98%. In all studies mentioned above ultrasound was not used in the radiological work-up of breast disease. Ultrasonography plays an important role in breast radiology. It should be performed as the initial imaging study in younger women with a palpable mass and the value of ultrasonographical guidance for interventional procedures is well established (Jackson, 1995). The use of ultrasonography will help to differentiate solid from cystic masses, and it frequently demonstrates a palpable mass that is not detected by mammography because of dense fibroglandular breast tissue (Sickles et al, 1984; Rosner and Blaird, 1985). Therefore, sensitivity and specificity will be increased by using ultrasonography complementary to mammography. Kaplan et al (1990) estimated a sensitivity of

**Table 5** Value of breast imaging according to the presenting symptoms / reason for breast imaging

| Presenting symptoms or reason for breast imaging | *n* | TP (*n*) | TN (*n*) | FP (*n*) | FN (*n*) |
|---|---|---|---|---|---|
| Screening (no symptoms) | 397 | 4 | 385 | 6 | 2 |
| Breast lump | 984 | 98 | 834 | 47 | 5 |
| Pain alone | 508 | 6 | 498 | 4 | 0 |
| Lumpiness with or without pain | 281 | 2 | 274 | 4 | 1 |
| Family history of breast cancer[a] | 251 | 2 | 249 | 0 | 0 |
| Follow-up after previous breast cancer | 370 | 4 | 361 | 2 | 3 |
| Nipple/skin problems | 104 | 4 | 99 | 0 | 1 |
| Other[b] | 119 | 18 | 99 | 2 | 0 |

[a]Breast cancer in at least one first-degree relative. [b]For example breast implants, follow-up of previous mammographic abnormality. TP, true positive radiological examination; TN, true negative; FP, false positive; FN, false negative.

© Cancer Research Campaign 1997

98% in a large series in which ultrasound was used complementary to mammography, but long-term follow-up was not available. This major shortcoming was also present in two other studies, in which a sensitivity of 97% was reported (Guyer, 1988; Den Heeten et al, 1993). Our study is the first one that estimates the sensitivity, specificity and predictive values of breast imaging using mammography and ultrasonography as integrated diagnostic modalities in a large number of prospectively identified, consecutive cases for which the follow-up data collection is virtually complete. We think that, because of the follow-up procedures applied and the complementary use of mammography and ultrasound, the values of the parameters obtained in our study are more in agreement with everyday reality than the results published in other series. The definition of a false-negative radiological examination used can markedly affect the sensitivity value obtained. Every mammographer knows of false-negative cases that were visible on a given study but went undetected, perhaps for several years. Unfortunately, there is no gold standard available with which the presence or absence of cancer can be determined unambiguously and thereby be used to measure the sensitivity of breast imaging. In screening programmes, interval cancers are cancers discovered between two screening examinations after a previous screening did not result in a request to perform a biopsy of the breast in which the cancer was subsequently found. Although some of these interval cancers may have arisen de novo between screenings, it seems unrealistic to assume that none was potentially detectable at screening. The other extreme is to assume that all of the cancers detected between screenings are false-negative cases, i.e. cases in which mammography fails to detect a proven cancer during the time of the trial. In several screening studies (Frisell et al, 1987; Peeters et al, 1989), 50–60% of the interval cancers are regarded as 'true' interval cancers (an obvious lesion is observed on the diagnostic mammogram while no suspect signs are seen on the previous screening mammogram). Our study does not concern a screening programme and therefore the term 'interval cancer' can not be used. For purposes of analysis, we considered all radiology reports of patients who developed breast cancer within 1 year after a negative radiological examination to be false negative. Negative radiology reports of patients initially presenting without clinical and radiological suspicion of cancer, and who after 1 year following these reports developed cancer, were considered in retrospect to be true negative.

A positive predictive value of 68.0% for the whole study population was found. The incidence of breast cancer was higher in the group referred for breast imaging by specialists. This difference is reflected in the higher positive predictive value for the patients referred by specialists (76% vs 60%). Comparison with other series is difficult as the positive predictive value of breast imaging depends on several other factors as well (Kopans, 1992). However, the integral use of mammography and ultrasonography will have helped us to obtain a positive predictive value that was substantially higher than in diagnostic studies in which ultrasound was not used (Wolfe et al, 1987; Robertson, 1993; Sienko et al, 1993).

It has been demonstrated that mammographic follow-up can be a safe alternative to biopsy in the cases of mammographically detected, probably benign lesions (De Neef and Gandera, 1991; Helvie et al, 1991; Sickles, 1991). In our study, 2% of patients (3 out of 143) with these lesions were shown to have breast cancer and the diagnostic delay in these cases was 6–8 months. One of these patients had positive axillary nodes, and we cannot assess whether the delay has compromised the outcome for this patient. On the other hand, additional pathological examination of all the probably benign lesions would have yielded an unacceptably low malignant–benign biopsy ratio. For these reasons we support the statement that radiological follow-up of probably benign lesions is a reasonable alternative to surgical biopsy.

Currently, various biopsy techniques are available for (non)palpable breast lesions. We determined the value of breast imaging and did not focus on additional biopsy techniques. In qualified hands, the FNAB (fine-needle aspiration biopsy) works reasonably well and open surgical biopsy can be avoided in many cases (Azavedo et al, 1989; Hindle et al, 1993). Recent studies suggest that core biopsy can be as accurate as open surgical biopsy in the work-up of (non)palpable lesions (Elvecroq et al, 1993; Parker et al, 1994). During the study period, core biopsy was not routinely performed at our hospital.

Five patients with a dominant lump and a negative radiology report were shown to have breast cancer. These cancers could have been diagnosed properly if representative pathological examination had been performed. Again, in our series, this approach would have resulted in a very low malignant–benign biopsy ratio.

One patient presented with breast cancer more than 18 months after a false-negative radiological test. As follow-up ranged between 18 and 42 months in our series, we are not certain that the minimum observation period of 18 months was sufficient for the detection of all false-negative cases. Therefore, the actual sensitivity and specificity might be slightly less than those calculated. The outcome of our study could be biased if radiological examination was not performed before surgery in a substantial proportion of the breast cancer patients. This would leave a relatively benign population for breast imaging. However, at our hospital, breast imaging is nearly always performed before possible pathological examination in accordance with state of the art work-up of symptomatic breast disease.

We conclude that breast imaging, consisting of the integral use of mammography and ultrasonography, is a valuable tool in the detection of cancer and should therefore be included in the work-up of symptomatic breast disease.

### REFERENCES

Azavedo E, Svane G and Auer G (1989) Stereotactic fine-needle biopsy in 2594 mammographically detected non-palpable lesions. *Lancet* **1**: 1033–1036

Baines CJ, Miller AB, Wall C, McFarlane DV, Simor IS, Jong R, Shapiro BJ, Audet L, Petitclerc M, Ouimet-Oliva D, Ladouceur J, Hebert G, Minuk T, Hardy G and Standing HK (1986) Sensitivity and specificity of first screen mammography in the Canadian National Breast Screening Study: a preliminary report from five centers. *Radiology* **160**: 295–298

Bassett LW, Kimme-Smith C, Sutherland LK, Gold RH, Sarti D and King W (1987) Automated and hand-held breast US: effect on patient management. *Radiology* **165**: 103–108

Bird RE (1989) Low-cost screening mammography: report on finances and review of 21,716 consecutive cases. *Radiology* **17**: 87–90

De Neef PD and Gandara J (1991) Experience with indeterminate mammograms. *West J Med* **154**: 36–39

Den Heeten GJ, van Rooij WJ and Roukema JA (1993) Ultrasonography important as a supplement to mammography. *Ned Tijdschr Geneeskd* **137**: 2378–2383

Egan RL and Egan KL (1984) Automated water-path full-breast sonography: correlation with histology of 176 solid lesions. *Am J Roentgenol* **143**: 499–507

Elvecroq E, Lechner MC and Nelson MT (1993) Non-palpable breast lesions: correlation of stereotaxic large-core needle biopsy and surgical biopsy results. *Radiology* **188**: 453–455

Fleischer AC, Muhletaler CA, Reynolds VH, Machin JE, Thieme GA, Bundy AL, Winfield AC and James AE Jr (1983) Palpable breast masses: evaluation by high frequency, hand-held real-time sonography and xeromammography. Work in progress. *Radiology* **148**: 813–817

© Cancer Research Campaign 1997

Fornage BD, Lorigan JG and Andry E (1989) Fibroadenoma of the breast: sonographic appearance. *Radiology* **172**: 671–675

Frisell J, Eklund G, Hellstrom L and Somell A (1987) Analysis of interval breast carcinomas in a randomized screening trial in Stockholm. *Breast Cancer Res Treat* **9**: 219–225

Guyer PB (1988) Direct-contact B-scan sonomammography – an aid to X-ray mammography. *Ultrasound Med Biol* **14** (suppl. 1): 49–52

Hansell DM, Cooke JC and Parsons CA (1988) The accuracy of mammography alone and in combination with clinical examination and cytology in the detection of breast cancer. *Clinical Radiol* **39**: 150–153

Harper AP, Kelly-Fry E, Noe JS, Bies J and Jackson VP (1983) Ultrasound in the evaluation of solid breast masses. *Radiology* **146**: 731–736

Helvie MA, Pennes DR, Rebner M and Adler DD (1991) Mammographic follow-up of low-suspicion lesions: compliance rate and diagnostic yield. *Radiology* **178**: 155–158

Hindle WH, Payne PA and Pan EY (1993) The use of fine-needle aspiration in the evaluation of persistent palpable dominant breast masses. *Am J Obstet Gynecol* **168**: 1814–1818

Jackson VP (1995) The current role of ultrasonography in breast imaging. *Radiol Clin N Am* **33**: 1161–1170

Kaplan C, Matallana R and Wallack MK (1990) The use of state-of-the-art mammography in the detection of nonpalpable breast carcinoma. *Am Surg* **56**: 40–42

Kopans DB (1992) The positive predictive value of mammography. *Am J Roentgenol* **158**: 521–526

Locker AP, Manhire AR, Stickland V, Caseldine J and Blamey RW (1989) Mammography in symptomatic breast disease. *Lancet* **1**: 887–889

Parker SH, Burbank F, Jackman RJ, Aucreman CJ, Cardenosa G, Cink TM, Coscia JL, Eklund GW, Evans III WP, Garver PR, Gramm HF, Haas DK, Jacob KM, Kelly KM, Killebrew LK, Lechner MC, Perlman SJ, Smid AP, Tabar L, Taber FE and Wynn RT (1994) Percutaneous large-core breast biopsy: a multi-institutional study. *Radiology* **193**: 359–364

Peeters PH, Verbeek AL, Hendriks JH, Holland R, Mravunac M and Vooijs GP (1989) The occurrence of interval cancers in the Nijmegen screening programme. *Br J Cancer* **59**: 929–932

Reintgen D, Berman C, Cox C, Baekey P, Nicosia S, Greenberg H, Bush C, Lyman GH and Clark RA (1993) The anatomy of missed breast cancers. *Surg Oncol* **2**: 65–75

Robertson CL (1993) A private breast imaging practice: medical audit of 25,788 screening and 1,077 diagnostic examinations. *Radiology* **187**: 75–79

Rosner D and Blaird D (1985) What ultrasonography can tell in breast masses that mammography and physical examination cannot. *J Surg Oncol* **28**: 308–313

Sickles EA (1991) Periodic mammographic follow-up of probably benign lesions: results in 3,184 consecutive cases. *Radiology* **179**: 463–468

Sickles EA, Filly RA and Callen PW (1984) Benign breast lesions: ultrasound detection and diagnosis. *Radiology* **151**: 467–470

Sickles EA, Ominsky SH, Sollitto RA, Galvin HB and Monticciolo DL (1990) Medical audit of a rapid-throughput mammography screening practice: methodology and results of 27,114 examinations. *Radiology* **175**: 323–327

Sienko DG, Hahn RA, Mills EM, Yoon-DeLong V, Ciesielski CA, Williamson GD, Teutsch SM, Klenn PJ and Berkelman RL (1993) Mammography use and outcomes in a community (The Greater Lansing area mammography study). *Cancer* **71**: 1801–1809

Standertskjold-Nordenstam CG and Svinhufvud U (1980) Mammography of symptomatic breasts: a report on 1119 consecutive patients. *Ann Chir Gynaecol* **69**: 48–53

Tabar L and Dean PB (1985) *Teaching Atlas of Mammography*. Georg Thieme: Stuttgart

Tabar L, Okerlund E and Gad A (1984) Five-year experience with single-view mammography randomized controlled screening in Sweden. *Recent Results Cancer Res* **90**: 105–113

Warwick DJ, Smallwood JA, Guyer PB, Dewbury KC and Taylor I (1988) Ultrasound mammography in the management of breast cancer. *Br J Surg* **75**: 243–245

Wolfe JN, Buck KA, Salane M and Parekh NJ (1987) Xeroradiography of the breast: overview of 21,057 consecutive cases. *Radiology* **165**: 305–311

© Cancer Research Campaign 1997